# Recent Progress in Phage Therapy to Modulate Multidrug-Resistant *Acinetobacter baumannii*, including in Human and Poultry

**DOI:** 10.3390/antibiotics11101406

**Published:** 2022-10-13

**Authors:** Yan Zhang, Yuanqing Lin, Salvatore Galgano, Jos Houdijk, Weiquan Xie, Yajie Jin, Jiameng Lin, Wuqiang Song, Yijuan Fu, Xiuying Li, Wenting Chui, Wei Kan, Cai Jia, Guangwei Hu, Tao Li

**Affiliations:** 1 Shanghai Veterinary Research Institute, Chinese Academy of Agricultural Sciences (CAAS), Shanghai 200241, China; 2 Animal Disease Prevention and Control Center in Qinghai Province, Xining 810001, China; 3 Monogastric Science Research Centre, Scotland’s Rural College, Roslin Institute Building, Edinburgh EH25 9RG, UK; 4 School of Pharmacy, Guilin Medical University, Guilin 541199, China

**Keywords:** multidrug-resistant *Acinetobacter baumannii*, phage therapy

## Abstract

*Acinetobacter baumannii* is a multidrug-resistant and invasive pathogen associated with the etiopathology of both an increasing number of nosocomial infections and is of relevance to poultry production systems. Multidrug-resistant *Acinetobacter baumannii* has been reported in connection to severe challenges to clinical treatment, mostly due to an increased rate of resistance to carbapenems. Amid the possible strategies aiming to reduce the insurgence of antimicrobial resistance, phage therapy has gained particular importance for the treatment of bacterial infections. This review summarizes the different phage-therapy approaches currently in use for multiple-drug resistant *Acinetobacter baumannii*, including single phage therapy, phage cocktails, phage–antibiotic combination therapy, phage-derived enzymes active on *Acinetobacter baumannii* and some novel technologies based on phage interventions. Although phage therapy represents a potential treatment solution for multidrug-resistant *Acinetobacter baumannii*, further research is needed to unravel some unanswered questions, especially in regard to its in vivo applications, before possible routine clinical use.

## 1. Introduction

The Gram-negative aerobic, non-motile, pleomorphic bacillus *Acinetobacter baumannii* [1] is a multidrug-resistant opportunistic pathogen, currently identified as one of the major causes of nosocomial infections in the healthcare system worldwide [2]. Moubareck et al. defined *A. baumannii* as the main causative agent of pneumonia, sepsis, meningitis and urinary tract and wound infections [3], with it being correlated to a nosocomial mortality rate of up to 35% [4]. Antimicrobial resistance (AMR) has been identified as a major worldwide health threat; in recent years, the irrational use of antibiotics, especially in broad-spectrum approaches, has led to both an increased selection of microbial species able to survive medical treatments and an increased genomic distribution of AMR genes [5,6]. Amongst the increasing number of multidrug-resistant bacteria reported, *A. baumannii* has also been correlated to an increased resistance to multiple antibiotics [7]. Different outcomes have been reported for *A. baumannii* infected patients, including the need for cardiac surgery, with a prevalence of high-mortality pulmonary infections [8]. According to Asif et al. (2018), *A. baumannii* AMR gene distribution significantly differs between patients in different hospitals and departments [9]. Specifically, *A. baumannii* has been found to be resistant to common antibiotics, such as cefoperazone/sulbactam, ampicillin/sulbactam and piperacillin/tazobactam, while polymyxin B still showed strong antibacterial activity against multidrug resistant *A. baumannii* in vitro [10]. *A. baumannii* infection and drug resistance rates are generally increasing, leading to a decrease in the effectiveness of general antibiotic therapy worldwide. For example, carbapenems are critically important broad-spectrum antibiotics, whose pivotal therapeutical role is endangered by the insurgence of multi-resistance amongst multidrug resistant *A. baumannii* [6]. There is increasing evidence that extensively drug-resistant (XDR) and pan-drug-resistant (PDR) *A. baumannii* strains accumulate in, amongst others, countries such as Iran and Croatia [11,12,13].

Poultry production has an essential contribution in terms of food security and nutrition, with a fast-growing market [14], mostly due to poultry meat and eggs being an affordable protein source [15]. A significant number of regulations have led to a decrease in the use of antimicrobial agents in food animal production [16]; however, *A. baumannii* is commonly found in poultry and their produce. Indeed, its role as a zoonotic AMR agent has been investigated [17], indicating the possible AMR transmission from poultry to humans [18]. Multidrug resistant *A. baumannii* has been listed as a key priority by the World Health Organization (WHO) in the attempt to identify pathogens that pose an increased threat to human health [19], hence the urgent need for alternative treatment strategies.

Bacteriophages (phages) are viruses that specifically target bacteria with a basic structure comprising an outer protein capsid enclosing the nucleic acid [20]. Similar to other viruses, a typical phage lytic infection cycle is characterized by adhesion to the bacterial cell via recognizing host outer receptors, the injection of a phage genome into the cytosol, viral replication, followed by bacterial lysis and the liberation of a new phage [21], which could potentially infect new susceptible bacterial cells. Phage therapy, based on such lytic dynamics, could function as a self-amplifying “drug”, targeting sensitive bacterial cells and therefore providing an alternative to antibiotic therapy [22]. Strictly speaking, lytic phages are usually preferred for phage therapy, whereas the use of temperate phages has been avoided due to their ability to mediate gene transfer between bacteria through specialized transduction, which may increase bacterial virulence [23] or horizontal AMR gene transfer [24]. Beyond being a promising alterative to classic antibiotics, with the aim of decreasing the insurgence of AMR, phages could be also used towards biofilms, with them having a lower systemic toxicity and improved self-reproduction abilities compared to classic antibiotics [25,26]. Phage therapy has been relatively poorly studied in the past, in contrast to the majority of the studies, which have focused their attention on classic antibiotics, targeting tolerance, immune response, pharmacokinetics, pharmacodynamics and animal models of infection [27]. Recently, a significant number of studies on phage therapy have been published, underlining the important role of this possible therapeutical alternative [4,27,28,29,30]. In 2017, phage therapy was reported as possible treatment for *A. baumannii* infection for the first time [31]. The advantages and limitations associated with phage therapy, as currently understood, are summarized in Table 1.

**Figure 1:**
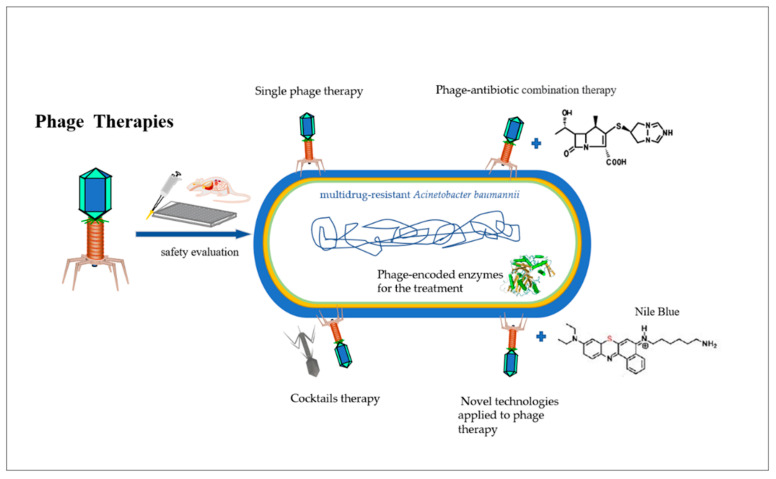
The most common phage therapies tested so far against pathogens, including single phage therapy, phage-cocktails, phage-antibiotic combination therapy, phage-encoded enzymes and novel approaches to phage therapy, such as its use in combination with photosensitizers.

Research on bacteriophages as an antibiotic alternative has become increasingly popular due to the rise of AMR and the increasing number of multi-drug-resistant bacteria. Numerous in vivo and in vitro studies using single or mixed phage types (phage cocktails) have been conducted over the years. The following section describes in detail the most common phage therapies tested so far, especially considering their applications against *A. baumannii* in both human medicine and applied to poultry production, including single phage therapy [45], phage-cocktails [46], phage-antibiotic combination therapy [47], phage-derived enzymes [48] and novel approaches to phage therapy, such as its use in combination with photosensitizers [49] (Figure 1).

## 2. Phage Therapy on Human Infection

### 2.1. Single Phage Therapy

Therapies based on a single virus type, also known as monophage therapies, have been extensively applied as *A. baumannii* treatments. Jeonet et al. (2012) found that the phage YMC 13/03/R2096 ABABBP or the molar φ-R2096 exhibited high lytic activity against *A. baumannii* growth in a dose-dependent manner [50]. In another study, intranasally administered phage SH-AB15519, originally isolated from hospital wastewater, was found to be effective in treating pneumonia led by carbapenem-resistant *A. baumannii* infection in mice [51]. Interestingly, phage SH-AB15519 has been demonstrated to be lacking genes connected to further virulence or AMR [22], possibly as a symptom of its low integration rate, which might endorse the use of this phage as a possible antibiotic alternative. PD-6A3 is a novel *A. baumannii* phage which also inhibits *Escherichia coli* and Methicillin-resistant bacteria [52]. Furthermore, Phage Abp9 effectively treated the biofilm produced by *A. baumannii* strain ABZY9 in vitro and contributed to positive treatment outputs in a murine model of *A. baumannii* infection [32]. Phage φ KM18P was used in XDR *A. baumannii* bacteraemia models in BALB/C and C57BL/6 mice, where it improved the survival rate of animals and reduced the number of bacteria in the blood, concurring with decreased levels of TNF- α and interleukin-6 [53]. The bacteriophage vB_AbaP_AGC01, isolated from a fish pond sample collected in Stargard (Poland), has been shown to have high specificity to *A. baumannii* and to generate high-yield viral offspring (317 ± 20 plaque-forming units per cell) [54]. The phage vB_AbaP_AGC01, either alone or in combination with antibiotics (gentamicin, ciprofloxacin and meropenem), significantly reduced *A. baumannii* cell counts in a human heat-inactivated plasma model [54]. In parallel, the phage vB_AbaM_PhT2 prevented *A. baumannii*-induced cell damage in human brain and bladder cell lines by significantly reducing bacterial cytotoxicity and the dose of colistin needed [55]. Therefore, these findings suggest that phages in general, and perhaps phage vB_AbaM_PhT2 in particular, could be applied as antibacterial agents in a hospital environment. The bacteriophage STP4-A, screened by Li et al., has a strong inhibitory effect on both single and multiple salmonella strains and is a safe antibacterial agent with a wide host range which can be used in the poultry industry. Tawakol et al. [56] showed that phage therapy (via intratracheal inoculation) not only reduced the severity of APEC infection when studied as a single pathogen infection, but also prevented mortality from the co-infection of APEC and infectious bronchitis virus (IBV). In addition, phage treatment significantly reduced the number of pathogenic exfoliated *E. coli* and IBV in the mixed infection group but not in the case of the IBV-only challenge.

### 2.2. Cocktail Therapy

Phage cocktails typically consist of multiple phages combined, with each of them having unique host specificity due to selective affinity towards a specific bacterial receptor, conferring a broad therapeutic lysis spectrum [57]. On the other hand, the development of phage resistance, especially to lytic viruses, should be carefully monitored, and cocktails appear to be a valid approach to limit such occurrences. It has been shown, for example, that a designed cocktail of the phages vB_AbaS_D0, isolated from hospital-sewage samples in Dalian (China), and vB_AbaP_D2 decreased the mutation frequency of *A. baumannii* whilst also decreasing the percentage of phage-resistance in a murine bacteraemia model [58]. Wu et al. reported the the administration of a phage cocktail (φAb121 and φAb124) to four patients in a COVID-19 intensive care unit in China was able to treat carbapenem-resistant *A. baumannii* infection, otherwise showing the insurgence of phage-resistant *A. baumannii* strains when only one phage was administered [59]. The application of a cocktail of bacteriophages has also been demonstrated to be an effective substitute to antibiotic growth promoter replacement in broiler diets [60], which would further assist in the reduction of the development of anti-microbial resistance arising from poultry production. The combination of phages (φkm18p, φTZ1 and φ314) as a cocktail was able to decrease the concentration of *A. baumannii* in another study, in contrast to single-phage administration, which was correlated to recidivist bacterial growth [61]. In parallel, another study demonstrated an improved outcome when using q phage cocktail compared to single phage in lysing *A. baumannii* bacteria without leading to further resistance [62].

Similarly, the emergence of anti-phage mutants can be suppressed by ensuring a high titre throughout cocktail treatment. Beyond phage-resistance, another factor to consider is that treatment with high-populated phage cocktails may lead to complex pharmacological and immune responses, which may hinder the implementation of clinical trials [44], hence the recommendation of the use of a less complex cocktail consisting of up to 2–10 phages as the first choice [63]. As observed in other fields, the misuse of antibiotics associated with livestock, including in poultry production, has led to the selection and spread of multi-drug resistant organisms (MDROs), including *A. baumannii* [64]. The zoonosis risk associated with these MDROs is not only clinically relevant to the development of a specific symptomatology, but it could also contribute to the spread of AMR to humans thanks to mechanisms such as, e.g., horizontal gene transfer. Although the use of phage therapy to control *A. baumannii* infection in poultry has not been reported, many studies have been carried out on other pathogens in farm animals. Indeed, Campylobacter jejuni abundance in broilers was decreased by oral treatment with a Campylobacter-specific- phage cocktail, without further affecting microbiota species [65], providing a working example for the further future application of similar strategies to modulate *A. baumannii* overgrowth in poultry and other livestock.

### 2.3. Phage–Antibiotic Synergy

Phage–antibiotic synergy (PAS) refers to the usage of antibiotics at sublethal doses in combination with phage administration, with the aim of increasing the release of phage-progeny from bacterial cells [66]. PAS strategies have a number of advantages, such as enhanced bacterial inhibition, the reduced development of phages and the penetration of biofilms [67]. However, care should be taken when considering a combined therapy due to their unavoidable increased risk towards AMR insurgence. Low antibiotic doses used in such combinations could indeed facilitate the selection of resistant species. Moreover, the impact of these antibiotics on the rest of the microbiota symbionts, beyond the primary target, ought to be taken into consideration [68].

Importantly, the final PAS effect is affected by not only the qualitative distribution of antibiotics in the mix, but also by their relative concentrations. Ma et al. [69]. optimized the multiplicity of infection (MOI, i.e., optimal phage/target ratio) of phages in combination with eight different antibiotics applied to the control of *A. baumannii*, demonstrating that a reduction in the rifampicin concentration led to a decreased PAS effect, which was otherwise increased by a decrease in both meropenem and minocycline concentrations. On the other hand, the effectiveness of PAS, as a combined approach, has been shown in several studies. Indeed, Grygorcewicz et al. observed approximately a 4-log reduction of *A. baumannii* when using vB_AbaP_AGC01 phage in combination to ciprofloxacin and meropenem, in a heat-inactivated plasma blood model [54]. Tan et al. reported pathogen clearance and clinical improvement in patients previously diagnosed with carbapenem-resistant *A.* *baumannii* pneumonia after treatment with monophage preparation in combination with tigecycline and polymyxin E [70]. The phage VB_ABam-Kar-1 characterized by Jansen et al. [71] showed lytic activity against clinical isolates of multidrug resistant *A. baumannii*. The latter was shown when the multiplicity of infection of kar-1 was 10^−7^ and the therapeutic dose of colistin and meropenem significantly increased the inhibition of bacteria. The synergy of phage–antibiotic therapies was measured by Grygorcewicz et al. [72], demonstrating different types of interactions between phages and antibiotics (i.e., synergistic, additive, indifferent or antagonist interactions) depending on the antibiotic used. For example, bacteriophage AGC-01 had a synergistic interaction with ciprofloxacin and norfloxacin, whilst vB_AbaP_AGC01 phage had an additive interaction with norfloxacin and meropenem, both on *A. baumannii*. Interestingly, when using *E. coli* as a test organism, it was found that phages could lower the minimum inhibitory concentration for drug-resistant strains and that the emergence of resistant cells was suppressed by the synergism between phages and antibiotics [73].

### 2.4. Phage-Encoded Enzymes for the Treatment of A. baumannii

#### 2.4.1. Endolysins

Endolysins are phage-produced hydrolases that lyse bacterial cell walls, allowing the further release of progeny phages at the end of the replication cycle [74]. These enzymes are very effective towards peptidoglycan layers, leading to a sudden drop in osmotic pressure and therefore lysis [75]. According to their action on the main bonds in the peptidoglycan layer, endolysins are divided into five categories: (I) N-acetyl-β-D-intracellular amidase, (II) N-acetyl-β-D-glucosaminidase, (III) transglycosidase; (IV) N-acetyl-leucoyl-l-alanine amidase and (V) L-alaninoyl-D-glutamate endopeptidase [76]. The main advantage of endolysin therapy over traditional broad-spectrum antibiotics is endolysins’ high specificity towards bacterial species or subspecies without interacting with the surrounding microbial cells [77]. Additionally, further advantages associated with endolysins are connected to reduced resistance, to their synergistic activity with different antibacterial agents and to their ability to play an effective role on biofilm and the mucosal surface [78].

TS2631, an endolysin from the Thermus scotoductus bacteriophage vB_Tsc2631, can also lysate *A. baumannii* and *P. aeruginosa* [79]. Wu et al. overexpressed and purified endolysin (Ply6A3) from vB_AbaP_PD-6A3, demonstrating its effectiveness towards 179 out of the 552 clinical multidrug-resistant *A. baumannii* strains tested (32.4%). In vitro, Ply6A3 not only inhibited *A. baumannii* but also other strains such as *E. coli* and MSRA, indicating Ply6A3 activity targeting the MSRA cell wall. During the observation period, no obvious side effects were observed after the intraperitoneal injection of Ply6A3 in mice [54]. In another trial, the activity profiles of recombinant endotoxins firstly identified and isolated from members of the Myoviridae phage family (LysAm24, LysAp22, LysECD7 and LysSi3) [80] were estimated to be effective towards one hundred Gram-negative pathogens, including the clinical isolates MDR Klebsiella pneumoniae, Salmonella, *P. aeruginosa*, *E. coli*, *A. baumannii* and *Enterobacter* spp. Of the bacteria investigated, *A. baumannii* was the most sensitive to endolysin. The data showed that these enzymes did not promote the development of short-term drug resistance. Furthermore, LysSi3 and LysECD7 did not decrease Bifidobacterium and Lactobacillus abundance in humans [81]. In addition, LysAB54 from *A. baumannii* bacteriophage p54 showed high antibacterial activity against a variety of Gram-negative pathogens [31]. Free peptidoglycan within the gastrointestinal tract is another endolysin target. In monogastric farm animals, and poultry in particular, peptidoglycan in bacterial cell debris may undermine gastrointestinal functionality. The supplementation of microbial muramidase with endolysin activity has been shown to benefit growth performance and gastrointestinal functionality in broilers [82,83,84]. With poultry being a reservoir for MDROs, the use of endolysin-based feed additives might assist in the reduction of the AMR level ending in the food chain.

#### 2.4.2. Depolymerases

During biofilm formation, bacterial cells are usually surrounded by extracellular polymers (EPSs), which can also act as barriers for phage penetration [85]. *A. baumannii* EPSs increases the resistance of the bacterium to antimicrobial agents due to diffusion limitation and can lead to severe persistent infections that are particularly difficult to treat, with them also providing resistance to phages [86]. Depolymerases are phage-derived enzymes that facilitates the early stages of phage infection by degrading extracellular bacterial protein [87]. The depolymerase responsible for degrading EPSs or O-polysaccharides can be found either as a virion component or it can be secreted in a soluble form during bacterial cell lysis [88]. This unique ability of depolymerases to specifically recognize and degrade EPSs and related biofilm components provides an attractive and promising tool for pathogen control [89]. On the other hand, biofilms are also known to develop within drinking lines in, e.g., poultry production systems (Maes et al., 2019), pointing towards the use of depolymerases as a management practice, with it also assisting AMR management. An example is provided by the tail spike protein derived from φAB6 with depolymerase activity, which can significantly inhibit the formation of and degrade existing biofilms at a concentration ≥0.78 ng [90]. Moreover, such proteins have also been found to be effective in reducing *A. baumannii* adhesion on the surface of medical devices [90].

### 2.5. Novel Technologies Applied to Phage Therapy

Recently, a number of technological developments based on phage therapy have been described, in addition to the traditional therapeutic schemes mentioned so far. One application is based on the work of Ran et al. (2021), who developed a unique photodynamic antimicrobial agent (APNB) based on a cationic photosensitizer and a bacteriophage for precise bacterial eradication, also showing high efficacy against biofilm [91]. NB is a benzoxazine compound, which is a well-known DNA-binding dye with relatively low systemic toxicity and in some cases is also known for delaying tumoral growth. In this context, NB can direct selective phototoxicity in combination to phage therapy, increasing the effectiveness of the latter, which when used alone could not achieve optimal therapeutic results [92]. The combination of the dye to the phage as an antimicrobial agent allows for the real-time monitoring and evaluation of the treatment dynamics, based on the NB fluorescence. Further structural modification with, e.g., sulphur atoms provide an excellent reactive oxygen species generation ability, which could be used in combination with APNB specificity towards binding pathogenic microorganisms. Both in vitro and in vivo experiments demonstrated that APNB can effectively treat *A. baumannii* infection. However, it ought to be mentioned that *A. baumannii* recovered faster after APNB treatment compared to ampicillin and polymyxin B in mice, despite APNB having promise with regard to its application against MDRP and biofilm [49].

In terms of new technologies based on phage therapy, aerosol spray applied to both poultry and bedding material in production facilities may help prevent the horizontal transmission of pathogens. Indeed, phage-based products can be used as biological disinfectants in hatcheries, farms, transport containers, poultry processing plants and food contact surfaces. Although not trialed against *A. baumannii*, bacteriophage-based surface disinfectants, such as BacWash TM (OmniLytics Inc., Salt Lake City, UT, USA), which targets Salmonella, can be used as cleaning agents. Similarly, Ecolicide PX™, which targets *E. coli* O157:H7, has been developed to purify the skin of live animals prior to slaughter [93]. El-Gohary et al. [94] demonstrated that treating pads by spraying a bacteriophage preparation against *E. coli* could limit its spread in broilers. Similar phage therapy applications are rarely reported against *A. baumannii*; however, based on these successful examples in poultry production, it is particularly important to study and include *A. baumannii* as a therapeutical target, both as a zoonotic agent and to limit the correlated spread of AMR.

## 3. Conclusions

Almost all newly developed antibiotics are variants of antibiotic classes discovered in the 1980s; however, the current reviewed and approved antibiotics inadequately address the challenges posed by the emergence and spread of AMR. Therefore, it is imperative to explore innovative approaches for the treatment of bacterial infections. Phage therapy represent an extremely promising, highly specific antimicrobial alternative. The phage mechanism of action relies on targeting and killing or inactivating sensitive bacteria specifically, and a variety of treatment options are under study at the moment, as described in this review, with implications not only for humans but also for poultry production. The latter is of specific importance, as it represents a reservoir for AMR and zoonotic bacteria. The efficacy and safety of phage therapy has been shown in the context of the treatment of multidrug-resistant *A. baumannii* through both in vitro and in vivo applications.

The current state of research on phage therapy is not comprehensive. Further clinical trials prior to successful routine applications in humans are important. In addition, several aspects of phage therapy require further elucidation, such as the stability of their formulation and industrial scaling, coupled with intrinsic caveats related to the possible insurgence of bacteriophage resistance, phage coevolution with bacteria and the broader effect on gut microbiota. Furthermore, other areas within this field may also need to be strengthened, such as for example the limited knowledge of the diversity of Acinetobacter phages. The latter is linked to the necessity of characterizing and classifying more phages active towards Acinetobacter. Another area needing further elucidation is the current scarcity of detailed knowledge in regard to the specific genes related to the pharmacodynamics of phages in the context of *A. baumannii* treatment, which will be certainly supported by the submission of whole-genome databases through the future years. There are reports for other bacterial species that suggest that phages carry antibiotic resistance genes or virulence determinants. A lack knowledge of the gene content of the phages employed as therapeutic agents may affect their performance and the associated outcome.

Prior to the routine application of phage therapy in medical practice, it is pivotal to select phages with limited negative effects on human tissues and interactions with the host-microbiota in order to limit both acute and chronic side effects. Moreover, there are currently caveats associated with phage delivery routes (i.e., oral, intravenous, intraperitoneal, subcutaneous, intramuscular, intranasal, intratracheal or topical), indicating the need to optimize the delivery method before the application of phage therapy in human medicine [95]. In parallel, the risk of phage-AMR needs to be evaluated and forecasted via applying opportune monitoring and modelling tools.

## Figures and Tables

**Table 1:** Advantages and limitations of phage therapy in comparison to antibiotics.

Advantage	Limitations
Narrow antimicrobial spectrum [32]	There is no definite optimal dosage and/or administration plan. Adaptive anti-phage immunity may develop through multiple dosing [33]
Abundant in water, soil and other ecological environment [34]	Technical challenges accompany the preparation of phagocytic mixture in advance [35]
Fewer side-effects [36]	Can promote horizontal gene transfer through transduction, which may lead to the spread of drug resistance [37]
Low environmental impact [32]	Lack of reproducibility amongst results from different in vivo and in vitro studies [38]
Low impact on broader microbial communities [39]	The immune response of the body may affect phage activity [40]
Low phage characterization and isolation cost [41]	Stability and shelf life [42]
Effective against bacterial biofilms [43]	Convoluted rational design (pharmacodynamics/pharmacokinetics) [44]
